# Regulation of microRNA expression by the adaptor protein GRB2

**DOI:** 10.1038/s41598-023-36996-3

**Published:** 2023-06-16

**Authors:** Amy K. Stainthorp, Chi-Chuan Lin, Dapeng Wang, Ragini Medhi, Zamal Ahmed, Kin Man Suen, Eric A. Miska, Adrian Whitehouse, John E. Ladbury

**Affiliations:** 1 School of Molecular and Cellular Biology and Astbury Centre for Structural Molecular Biology, University of Leeds, Leeds, LS2 9JT UK; 2 LeedsOmics, University of Leeds, Leeds, LS2 9JT UK; 3 Wellcome Centre for Human Genetics, University of Oxford, Oxford, OX3 7BN UK; 4 National Heart and Lung Institute, Imperial College London, London, SW3 6LY UK; 5 Wellcome Trust Cancer Research UK Gurdon Institute, University of Cambridge, Tennis Court Road, Cambridge, CB2 1QN UK; 6 Department of Molecular and Cellular Oncology, University of Texas MD Anderson Cancer Center, Houston, TX 77030 USA

**Keywords:** Biochemistry, Molecular biology

## Abstract

Protein interactions with the microRNA (miRNA)-mediated gene silencing protein Argonaute 2 (AGO2) control miRNA expression. miRNA biogenesis starts with the production of precursor transcripts and culminates with the loading of mature miRNA onto AGO2 by DICER1. Here we reveal an additional component to the regulatory mechanism for miRNA biogenesis involving the adaptor protein, growth factor receptor-bound protein 2 (GRB2). The N-terminal SH3 domain of GRB2 is recruited to the PAZ domain of AGO2 forming a ternary complex containing GRB2, AGO2 and DICER1. Using small-RNA sequencing we identified two groups of miRNAs which are regulated by the binding of GRB2. First, mature and precursor transcripts of mir-17~92 and mir-221 miRNAs are enhanced. Second, mature, but not precursor, let-7 family miRNAs are diminished suggesting that GRB2 directly affects loading of these miRNAs. Notably, the resulting loss of let-7 augments expression of oncogenic targets such as RAS. Thus, a new role for GRB2 is established with implications for cancer pathogenesis through regulation of miRNA biogenesis and oncogene expression.

## Introduction

The silencing of targeted genes by microRNA (miRNA) interference is a fundamental regulatory mechanism in eukaryotic cells^1^. The biogenesis of miRNA comprises multiple stages; i.e. primary miRNA (pri-miRNA) is initially transcribed then cleaved to produce precursor miRNA (pre-miRNA), followed by processing and then loading of mature miRNA onto an Argonaute (AGO) protein. AGO is incorporated at the core of the RNA-induced silencing complex (RISC). AGO alone may be sufficient for correct miRNA-loading^2,3^, but the efficiency of this process is improved through formation of the RISC-loading complex (RLC)^4^_._ In this complex, AGO directly binds the guide RNA-generating enzyme DICER1^5^.

**Figure 1:**
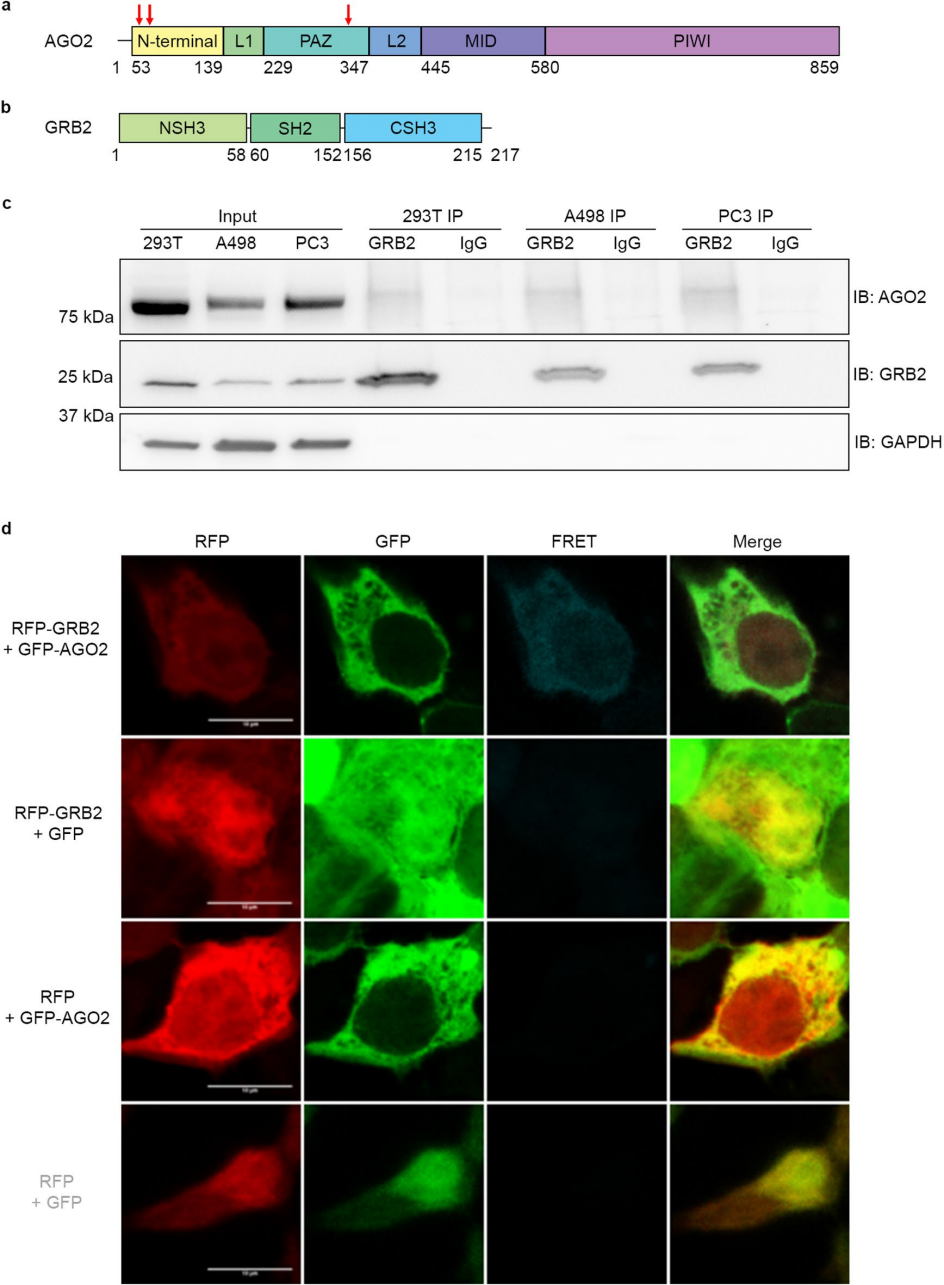
GRB2 complexes with AGO2 under non-stimulated conditions. Schematic diagram of, (**a**) AGO2 and, (**b**) GRB2 domain structures. Domains are named and colour coded and attributed amino acid sequence number. Red arrows indicate positions of PXXP motifs investigated in this work. (**c**) Western blot of AGO2 co-immunoprecipitated with GRB2 in serum starved HEK293T, A498 and PC3 cells. A longer exposure was used to capture AGO2 bands than for GRB2 and GAPDH. All images are taken from the same western blot. (**d**) Fluorescence and fluorescence resonance energy transfer signals of RFP-tagged GRB2 and GFP-tagged AGO2. HEK293T cells overexpressing fluorescent proteins were serum-starved before imaging. N = 3. Scale bars are 10 μm.

Of the four AGO proteins expressed in humans (AGO1-4), AGO2 is the most widely investigated due to its endonuclease activity^6,7^. AGO2 possesses six structured domains: N-terminal, L1, MID, L2, PAZ and PIWI (Fig. 1a). These domains form a two lobe structure across which the miRNA is bound, with the MID and PAZ domains anchoring the 5’ and 3’ ends, respectively^8–10^. The L1 and L2 domains help to maintain this structure^11^. In addition to its endonuclease activity, the PIWI domain is the recruitment site for several of the RISC proteins and DICER1^5,8,12^. The N-terminal domain is thought to be involved in passenger ejection^13^.

AGO2 is highly regulated by signalling proteins through both post-translational modification and protein–protein interactions. Phosphorylation of AGO2 serine S387 by AKT3 and MEK kinases regulates AGO2 activity and localisation^14–17^. EGFR-mediated phosphorylation of tyrosine Y393 is reported to occur under hypoxia and controls loading of miRNAs onto AGO2^18^. Y393 along with Y529 and Y749 can also be phosphorylated by SRC, which is known to inhibit guide RNA-binding^19,20^. Cycling phosphorylation and dephosphorylation of a C-terminal cluster of threonine and serine residues (S824–S834) is facilitated by casein kinase 1 alpha 1 (CSNK1A1) and ankyrin repeat domain 52 (ANKRD52)-protein phosphatase 6 (PP6), and is required for efficient miRNA-mediated gene silencing^21,22^. Most recently, ERK-mediated phosphorylation and glycosylation of AGO2 is proposed to facilitate interaction with E-cadherin to enhance RISC stability^23^.

The small GTPase RAS has been shown to regulate AGO2 unwinding of miRNAs in a mechanism unrelated to its catalytic function through an interaction with the AGO2 N-terminal domain^24^. AGO2 has also been proposed to interact with the adaptor protein GRB2 as a result of increased GRB2 expression in parasite-infected macrophages, and this is suggested to recruit the tyrosine phosphatase PTP1B to control miRNA-loading via Y393^25^. However, the mechanism of GRB2 binding AGO2 was not elucidated nor shown to be direct.

GRB2 is a highly conserved cytoplasmic protein which plays a multifunctional role in cells despite its lack of intrinsic enzyme activity^26^. GRB2 is composed of a central SRC homology 2 (SH2) domain flanked by N-terminal and C-terminal SRC homology 3 (SH3) domains (NSH3 and CSH3 respectively: Fig. 1b), which function to bind phosphorylated tyrosine residues and proline-rich motifs (PRMs), respectively^27–29^. GRB2 exists in a monomer–dimer equilibrium (*K*_D_ ~ 0.7 µM), however phosphorylation of GRB2 by an activated receptor tyrosine kinase (RTK) abrogates dimer formation^30,31^.

GRB2 is most often reported in its role of linking activated RTKs to downstream signalling effector proteins. As such, it is a key component of both the RAS and phosphatidylinositol 3-kinase (PI3K)/AKT signalling pathways^32,33^. Additionally, the ubiquitous cellular distribution of GRB2 allows it to play other functional roles (e.g., induction of conformational change to activate SHP2 phosphatase^34^ or promotion of homology-directed repair of DNA^35^). We have previously demonstrated that, in the absence of growth factor stimulation, the binding of GRB2 to FGFR2 via its C-SH3 domain regulates receptor-mediated signalling^30^. In this study, we show that under similar non-stimulated conditions GRB2 NSH3 directly binds to a PRM in the AGO2 PAZ domain. Through this complex GRB2 interacts indirectly with DICER1. Depletion of GRB2 in growth factor-deprived HEK293T cells dysregulates miRNA expression by two different mechanisms: (1) the 17~92 cluster and mir-221 family are diminished as both pri/pre and mature miRNA, and (2) let-7 family miRNAs are enhanced as mature transcripts only, suggesting regulation of miRNA loading or turnover. Concurrently, expression of oncogenic let-7 targets is repressed. Therefore, these data suggest a previously unknown role for GRB2 in the control of miRNA expression which is likely to have implications in tumorigenesis.

## Results

### The N-terminal SH3 domain of GRB2 associates with AGO2 PAZ domain under non-stimulated conditions

To investigate the complex formation between AGO2 and GRB2 we initially employed immunoprecipitation and GST-pulldown experiments using a variety of cell lines which had been serum-starved overnight. In the three cell lines tested AGO2 co-immunoprecipitated with GRB2 (Fig. 1c) and bound GST-tagged GRB2 (Extended Data Fig. 1). Complex formation was further confirmed upon overexpression of (red fluorescent protein) RFP-tagged GRB2 and (green fluorescent protein) GFP-tagged AGO2 in serum-starved HEK293T cells using fluorescence resonance energy transfer (FRET) (Fig. 1d).

**Figure 2:**
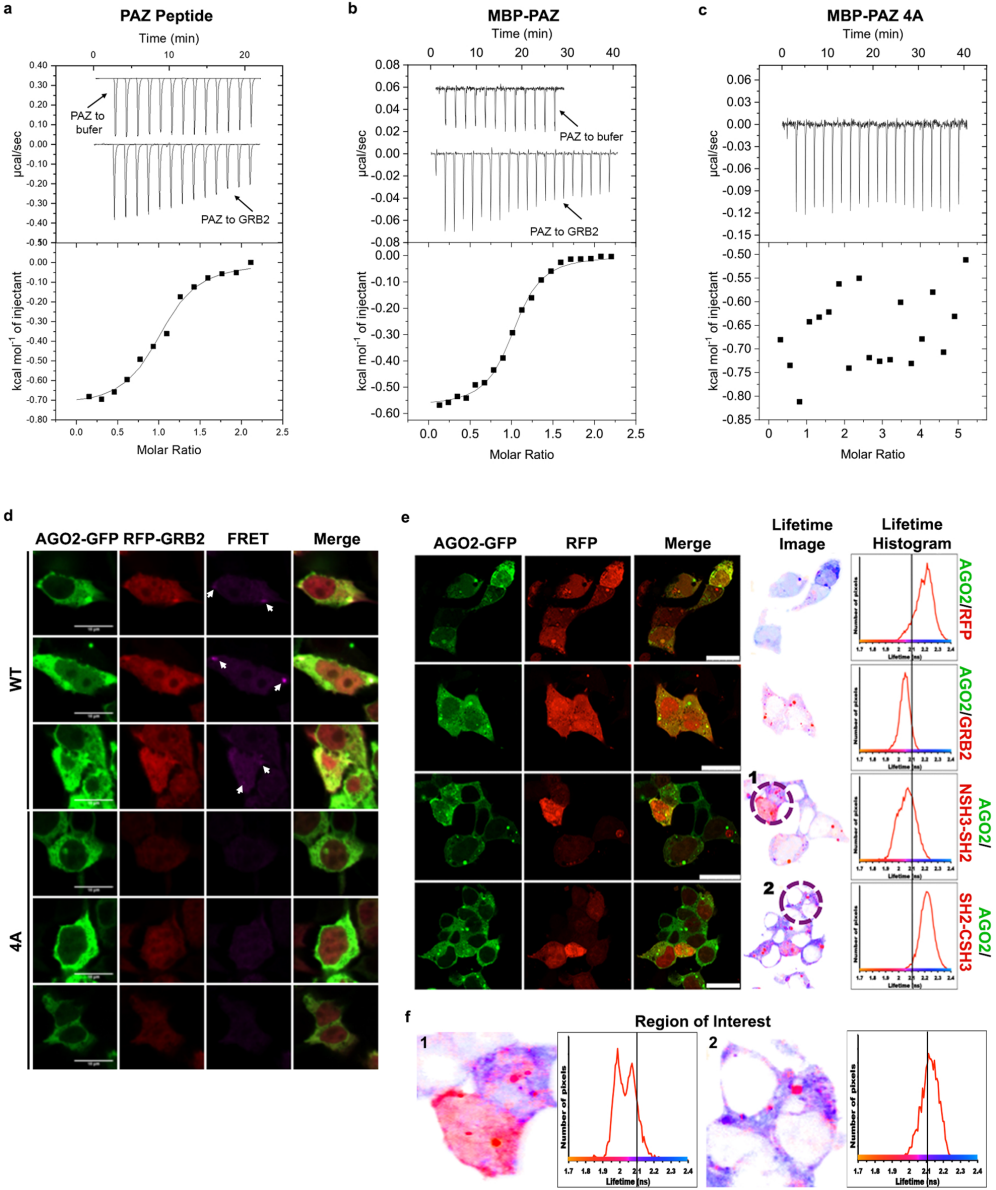
Binding of GRB2 to AGO2 is mediated by GRB2 NSH3 and a PXXP motif in AGO2 PAZ domain. (**a**) Isothermal titration calorimetry (ITC) of a peptide spanning the proline-rich motif ^323^PHLP^326^ in AGO2 PAZ domain. (K_D_ = 4.27 ± 1.17 µM). (**b**, **c**) ITC of MBP-tagged AGO2 PAZ domain titrated into GRB2. (**b**) PAZ WT (K_D_ = 585 ± 61 nM). (**c**) No binding observed for mutation of PXXP (MBP-PAZ 4A). N = 2. (**d**) Fluorescence resonance energy transfer (FRET) between wild type (WT) and ^323^AAAA^326^ (4A) mutant GFP-tagged AGO2 and RFP-tagged GRB2 in HEK293T cells under conditions of serum starvation. White arrows indicate intracellular puncta which show increased FRET when WT AGO2 is expressed. N = 2. Scale bars are 10 μm. (**e**) Fluorescence lifetime imaging microscopy of RFP-tagged GRB2 proteins and GFP-AGO2 overexpressed in serum-starved HEK293T cells. The formation of a protein complex results in a reduction in fluorescent lifetime represented by a shift to the left of the population of fluorophores (measured in number of pixels). Lifetime population distribution shown by red line on graphs. x = Lifetime (ns), y = number of pixels. Solid black line corresponds to average fluorescent lifetime for GFP, 2.1 ns. Scale bars 25 μm. (**f**) Expanded region of interest (ROI) further exemplifying left-shift for AGO2/NSH3-SH2 interaction.

The direct interaction between AGO2 and GRB2 was confirmed using isothermal titration calorimetry (ITC). Titration of the four MBP-tagged AGO2 domains into GRB2 revealed that only the PAZ domain bound GRB2 (Fig. 2a–c, Extended Data Fig. 2a–d). The binding isotherm could be fitted to a model with stoichiometry of one and a relatively tight affinity (*K*_D_ = 585 ± 61 nM: Fig. 2b). Since GRB2 exists as a dimer under the experimentally adopted concentration (20 μM)^30,31^, our data suggest that a heterotetramer including two PAZ domains and the GRB2 dimer could prevail.

Since our experiments were performed under basal conditions the binding between GRB2 and AGO2 occurs in a state of reduced cellular tyrosine phosphorylation. Under these conditions the binding of GRB2 is likely to be mediated via either of its SH3 domains binding to a PRM on cognate ligands rather than its SH2 domain binding to a phosphorylate tyrosine residue. AGO2 possesses three PRMs: two in the N-terminal region and one in the PAZ domain (Extended Data Table 1). Using ITC we found that GRB2 bound only to a PRM-containing peptide from the PAZ domain (residues 317–333, KLVLRYPHLPCLQVGQE: *K*_D_ = 4.3 ± 1.2 μM: Fig. 2a, Extended Data Fig. 3a, b) strongly suggesting that GRB2 is recruited to the AGO2 PRM ^323^PHLP^326^. The affinity is comparable to that of other SH3 domain-PRM interactions^36^. The SH3 domain-mediated direct interaction with the ^323^PHLP^326^ motif was further supported through binding studies incorporating mutation of AGO2 ^323^PHLP^326^ (Fig. 1a) in which substitution of the proline by alanine residues in MBP-PAZ (MBP-PAZ 4A) abrogated binding to GRB2 (Fig. 2c). The ^323^PHLP^326^ PRM is surface exposed, suggesting it is available to bind GRB2 and is not required for folding of PAZ (Extended Data Fig. 4a). In support of this, circular dichroism spectra of the wild type (WT) and 4A mutant domain indicated that PAZ largely remains folded when this PRM is mutated (Extended Data Fig. 4b).

**Table 1:** Affinity of AGO2 with DICER1 upon pre-incubation with GRB2. *K*_D_ was measured by microscale thermophoresis.

GRB2 concentration (µM)	*K*_*D*_ (µM)
0	17.6 ± 1.7
25	7.9 ± 1.3
50	4.6 ± 0.6

**Figure 3:**
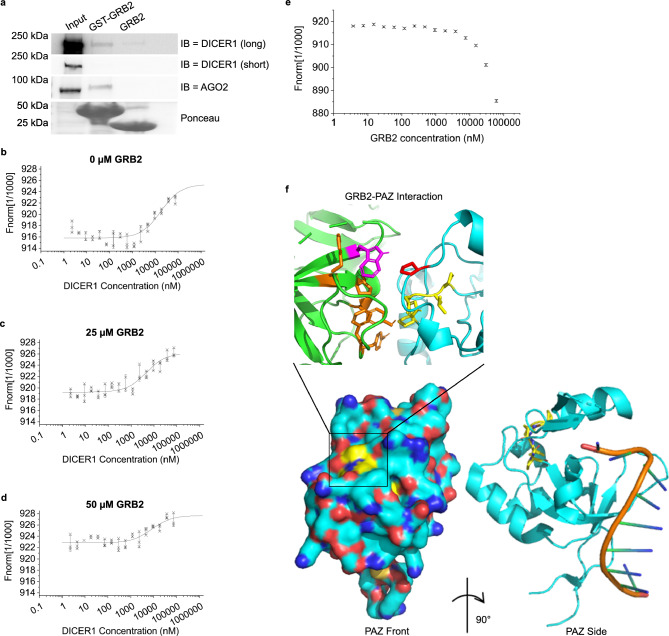
Impact of GRB2-AGO2 complex on interaction with DICER1 and miRNA. (**a**) Western blot of AGO2 and DICER1 pulldown by GST-GRB2 in HEK293T cells. HEK293T cells were serum-starved before lysis. Bands captured with both a long and short exposure are shown for DICER1, whereas only the image captured with a short exposure is shown for AGO2. GST proteins were detected by ponceau stain. All images are taken from the same western blot. N = 3. (**b–d**) MST of AGO2 binding to DICER1 C-terminal region, upon pre-incubation of AGO2 with increasing concentrations of GRB2. The difference in binding affinity was negligible. (**e**) MST of GRB2 with DICER1 C-terminal region. No binding is observed within a physiologically relevant range hence the two do not interact directly. (**f**) Expanded ribbon model of molecular docking of GRB2 (green; PDB: 1GRI^77^) to AGO2 PAZ domain (cyan; red and blue indicate positive and negative charges respectively; PDB: 6RA4^78^). The ^323^PHLP^326^ sequence is shown (yellow). GRB2 W36 (magenta) interacts with AGO2 P249 (red). Other residues in GRB2 which may contribute towards the interaction are shown in orange. Also shown is space-filling representation of AGO2 PAZ domain with PRM shown (below); and ribbon model of PAZ domain rotated by 90° to highlight juxtaposition of GRB2 binding site PRM and docking site for miRNA (right). Figures generated using PyMOL.

**Figure 4:**
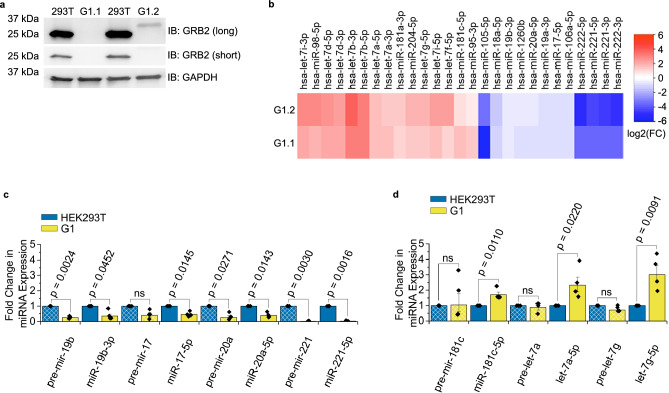
GRB2 regulates miRNA expression in HEK293T cells. (**a**) Western blot of GRB2 expression in wild type (293 T) and depleted (G1) HEK293T clones 1 (G1.1) and 2 (G1.2). While G1.1 is a complete knockout, G1.2 contains a deletion and large insertion in the N-terminal SH3 domain. GRB2 was blotted with an antibody which recognised the C-terminal SH3 domain. Both long and short exposures were used to capture the GRB2 bands, whereas the GAPDH image was captured using a short exposure only. All images are taken from the same western blot. N = 3. (**b**) Heat plot highlighting miRNAs which show significant log2(fold changes) in expression (p < 0.05) between wild type HEK293T and G1 cells, measured by small RNA sequencing. Cells were deprived of growth factor. miRNAs demonstrated positive (red) and negative (blue) expression changes. N = 2. (**c**, **d**) RT-qPCR analysis of fold-change in mean expression of precursor miRNA transcripts (precursor and primary, pre-mir-, hashed bars) and mature miRNA (miR-, plain bars) derived from serum-starved G1 or wild type HEK293T cells. Two groups of miRNAs were observed: (**c**) miRNAs which diminished at both the level of the precursor and mature transcripts and, (**d**) miRNAs which were enhanced as mature transcripts but not as precursors. Comparisons were made using a two-tailed Student’s t-test and error bars show standard error of mean. N = 4. ns = not significant.

To evaluate the role of ^323^PHLP^326^ in the context of the entire AGO2 protein, we mutated these four residues to alanines in GFP-tagged full length AGO2. Upon overexpression of this protein with RFP-tagged GRB2 in serum-deprived HEK293T cells, FRET was diminished in comparison to that with WT AGO2, suggesting that this motif is required for binding to GRB2 (Fig. 2d). Particularly apparent was the absence of intracellular puncta containing increased FRET signal.

To determine which GRB2 domain was bound by AGO2 we introduced PXXP-binding incompetent mutations in the NSH3 and CSH3 domains (W36K and W193K; RFP-GRB2^W36K^, RFP-GRB2^W193K^ respectively)^37^ and a pY-binding deficient SH2 domain mutation (R86A)^38^ into RFP-GRB2 (RFP-GRB2^R86A^). Overexpression of RFP-GRB2^R86A^ and RFP-GRB2^W193K^ with GFP-AGO2 in serum-starved HEK293T cells produced a FRET signal comparable to WT GRB2, whereas FRET was reduced in cells expressing the W36K mutant (as demonstrated by diminished fluorescence in column three: FRET, Extended Data Fig. 5). Consequently, since binding is depleted in the presence of the W36K mutant and is not impeded in the case of the mutated forms of the CSH3 and SH2 domains, our data suggest that AGO2 is recruited to GRB2 NSH3 but not to the CSH3 nor SH2 domains. To validate this observation, we used fluorescence lifetime imaging microscopy (FLIM) in non-stimulated HEK293T cells transfected with intact AGO2-GFP and one of the following: an empty RFP vector (negative control), full length GRB2-RFP, NSH3-SH2-RFP or SH2-CSH3-RFP (Fig. 2e). A decrease in the fluorescence lifetime of GFP indicates complex formation between GFP- and RFP-tagged proteins. This is clearly visible for the cells with intact GRB2 and the NSH3-containing construct. The region of interest (ROI) lifetime images from Fig. 2f, highlights the AGO2-NSH3 domain interactions in individual cells where the shift to shorter lifetime is pronounced in the case of the NSH3-SH2 construct. This is further exemplified in some of the intracellular puncta (red) seen in the Fig. 2f: ROI image 1.

**Figure 5:**
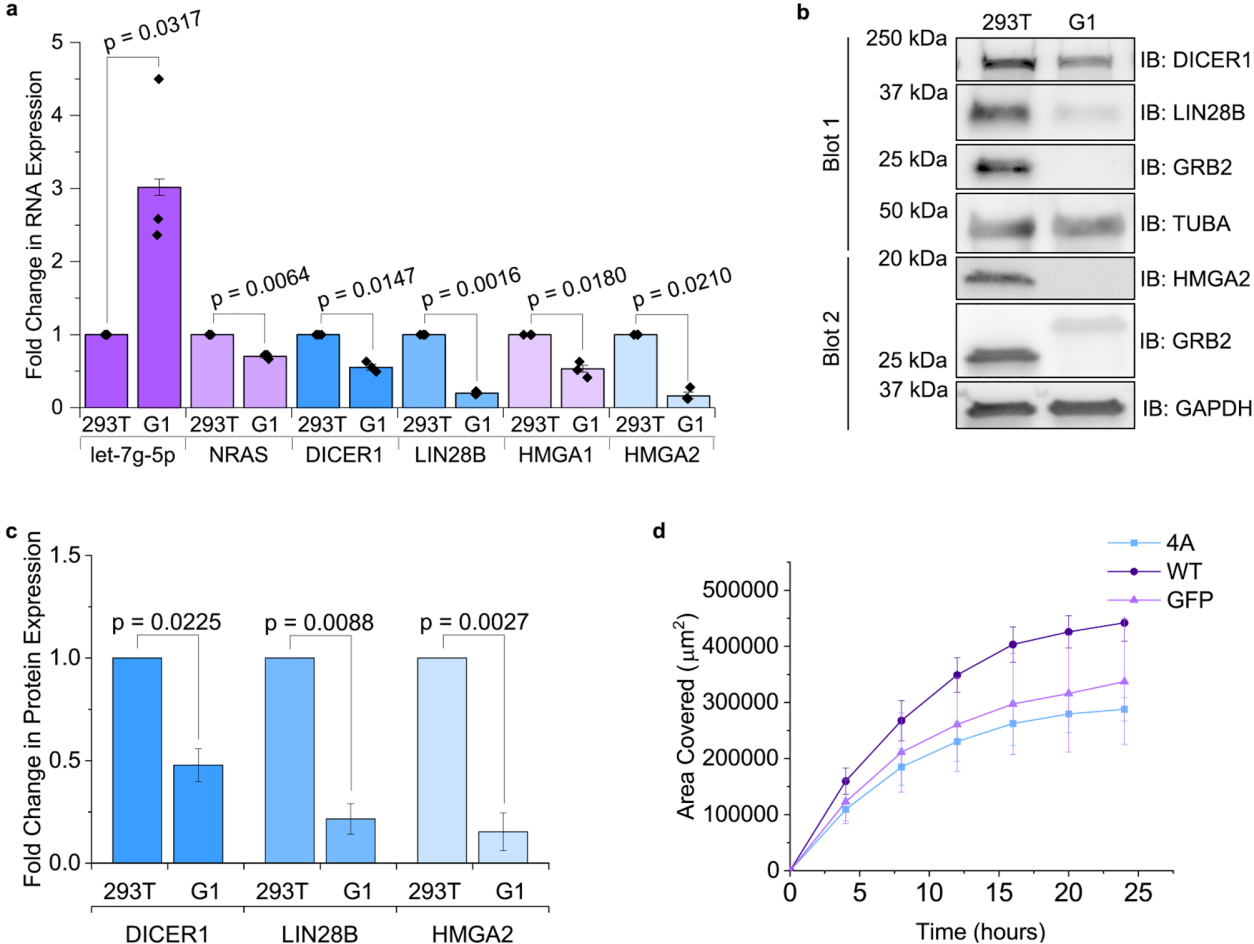
The GRB2-let-7 axis regulates oncogene expression. (**a**) RT-qPCR measurement of fold change in mean expression of let-7 g-5p miRNA and five target mRNAs in serum-starved GRB2 knockout cells (G1) compared to wild type HEK293T (293 T). Comparisons were made using a two-tailed Student’s t-test and error bars show standard error of mean. N = 3. (**b**) Western blot and (**c**) quantification of mean protein expression of let-7 targets in growth-factor-deprived G1 and HEK293T cells. The higher molecular band detected by the GRB2 antibody in G1 corresponds to an NSH3-mutated GRB2 polypeptide. For blot 1, a longer exposure was used to capture the DICER1 and GRB2 bands than was used for LIN28B and α-Tubulin. For blot 2, HMGA2 bands were captured using a longer exposure than that required for GRB2 and GAPDH. (**d**) Quantification of the area covered by migration of HEK293T cells expressing GFP-tagged wild type AGO2 (WT) or an AGO2 mutant which is incapable of binding GRB2 (4A), under conditions of reduced growth factor. Comparisons were made using a two-tailed Student’s t-test and error bars show standard error of mean. N = 3.

### The impact of GRB2-AGO2 complex on DICER1 and miRNA binding

Having established that GRB2 forms a direct interaction with AGO2 we investigated whether this interaction could prevail whilst AGO2 was involved in higher order functional complexes, such the RISC or RLC, which include the endoribonuclease DICER1. We immobilised GST-GRB2 on beads and incubated it with lysates from growth factor-deprived HEK293T cells. AGO2 and DICER1 were simultaneously pulled down by GST-GRB2 (Fig. 3a), suggesting the three proteins may exist in a complex together.

Incorporation of GRB2 into a complex with AGO2 and DICER suggests that it could play a functional role in the regulation of DICER1-mediated miRNA loading. Therefore, to investigate whether the formation of the GRB2-AGO2 complex regulates DICER1 binding to AGO2 we used microscale thermophoresis, MST. The interaction of AGO2, which had been incubated with increasing concentrations of GRB2, with the C-terminal domain of DICER1 (DICER1^CT^, residues 1276–1922 which contains the RNase III domains responsible for binding AGO2^39^) was measured. The *K*_D_ of DICER1^CT^ for AGO2 in the absence of GRB2 was 17.60 ± 1.67 µM and did not significantly change upon pre-incubation of AGO2 with GRB2 (Fig. 3b-d, Table 1) suggesting that binding of GRB2 to AGO2 does not impact on the AGO2-DICER1 interaction. Furthermore, the independent binding of GRB2 to DICER1^CT^ was found to be negligible (i.e. > 0.5 mM: Fig. 3e). However, whilst GRB2 does not bind DICER1^CT^, we cannot exclude the possibility of a GRB2 interaction with the N-terminal region, which contains several PRMs. Despite this possibility our data fit with a compelling and intuitive model whereby GRB2 and DICER1 bind independently at the PAZ and PIWI domains of AGO2 respectively.

The AGO2 PAZ domain is known to dock onto the 3’ end of the miRNA^3^. Thus, while association of GRB2 with AGO2 PAZ did not affect the DICER1 interaction, it may regulate binding of miRNA. To investigate the structural basis of the GRB2-AGO2 interaction and its proximity to the miRNA-binding site, a model of the GRB2-AGO2 complex was determined using an in silico docking experiment via HADDOCK2.2^40^. Docking of GRB2 NSH3 to AGO2 PAZ was facilitated by definition of the active residues involved in binding as being derived from the hydrophobic binding pocket of GRB2 NSH3^41^, i.e. Y7, F9, W36, F47, P49, and Y52. A model of GRB2 bound to the ^323^PHLP^326^ sequence of PAZ was successfully produced (Fig. 3f), defined by a large binding interface across GRB2 NSH3. The characteristic flat and amorphous binding surface (i.e., does not contain defined regions of charge that can be identified as cognate binding sites) of the NSH3 of GRB2 forms a platform for the surface-exposed AGO2 PAZ PRM which adopts a PPII helix located at the end of the miRNA-binding pocket. Consequently, it is possible that positioning of GRB2 in complex with AGO2 is able to regulate binding of the miRNA. Unexpectedly, W36 forms an additional interaction with P249 in the PAZ domain interface.

### GRB2 knockout inhibits expression of oncogenic miRNAs and promotes expression of tumour suppressor miRNAs

The formation of a ternary complex between GRB2, AGO2 and DICER1, three key protein components of the RLC, suggests a potential role for GRB2 in regulation of miRNA biogenesis. To explore this possibility, we used CRISPR/Cas9 to mutate *GRB2* in HEK293T cells and selected two clones: (1) with GRB2 depletion and (2) with abrogated NSH3 function. Genotyping and western blotting confirmed the complete knock out of GRB2 (clone G1.1) or a low level of expression of a mutated GRB2-derived polypeptide including an insertion in the NSH3 sequence, GRB2^ΔNSH3^ (clone G1.2, see Methods Fig. 4a). Since we know that the NSH3 domain binds to AGO2, clone G1.2 (as with G1.1) should not be able to sustain the GRB2-AGO2 interaction. Using G1.1 and G1.2 cells we investigated the effect of GRB2 on miRNA expression. In a small RNA sequencing study, miRNA expression in each clone was compared to both control HEK293T cells and to each other in the absence of growth factor. Our analysis identified a cohort of miRNAs that were dysregulated in cells depleted of GRB2 (Fig. 4b). These miRNAs demonstrated similar log2 (fold change) for both clones, confirming that they were not affected by expression of GRB2^ΔNSH3^. We observed that members of the same miRNA families were deregulated in a similar manner: mir-221, mir-17 and mir-19 were all diminished whereas let-7 and mir-181 were both enhanced in GRB2 depleted cells (Fig. 4b). Intriguingly, the downregulated miRNAs are known for their oncogenic properties^42,43^, whereas the let-7 family function as tumour suppressors^44^.

We performed independent qPCR and confirmed four downregulated, and three upregulated miRNAs identified in the sequencing study (Fig. 4c, d). Two miRNAs which were not identified in the miRNA screen were similarly not dysregulated by qPCR (Extended Data Fig. 6). Our qPCR was designed to investigate the stage at which miRNA biogenesis was perturbed. Thus, precursor miRNA expression was simultaneously quantified alongside that of the mature miRNA using primers which recognised both precursor and primary transcripts. pri- and pre-miRNA are produced early in the miRNA biogenesis pathway, whereas DICER1-mediated processing and loading of the miRNA onto AGO2 produces the mature transcript. pri/pre-forms of the miRNAs downregulated upon GRB2 depletion were reduced to a similar extent as their mature counterparts (Fig. 4c), suggesting both primary and precursor miRNAs were diminished. Thus, the stage at which expression was affected was likely to be before pri-miRNA cleavage, i.e., it was likely that these miRNAs were transcriptionally regulated. However, it has been shown production of pre-miRNA first requires processing of pri-miRNA to an intermediate product, suggesting that other steps may be involved^45^.

In contrast, expression of the pri/pre transcripts for miRNAs upregulated upon GRB2 depletion was similar in both G1 and HEK293T (Fig. 4d). Altered expression of mature, but not precursor, miRNA transcripts suggested that upregulation of these miRNAs was not mediated by enhanced DROSHA or DICER1 cleavage of precursor transcripts. Instead, it was likely that dysregulation occurred later in the biogenesis pathway and may have indicated changes in AGO2-loading, passenger strand degradation or miRNA turnover. However, as both -5p and -3p arms of the mature let-7 miRNAs were augmented (Fig. 4b) it seemed unlikely that passenger strand degradation was modified.

To confirm that the altered levels of miRNA expression reflected a change in miRNA-loaded AGO2, we immunoprecipitated AGO2 from serum-starved G1 and HEK293T cells and measured binding of let-7 by qPCR. The experiment was carried out under native conditions, i.e., without crosslinking, and although existing RNA–protein complexes should be maintained it is possible for complexes to reassociate after lysis. Indeed, Ct values in both control and test experiments were variable, reflecting the nature of these interactions as highly sensitive to perturbation during this experiment. Therefore, we believe that in this case the commonly chosen significance level of p < 0.05 may be too stringent and it may be more appropriate to apply a significance level of p < 0.1. AGO2-associated let-7a-5p was enhanced in G1 cells (p = 0.0827, Extended Data Fig. 7), supporting our theory that GRB2 regulates overall levels of let-7-loaded AGO2.

GRB2 is known to control protein tyrosine phosphorylation and kinase activity^24^. Equally, tyrosine phosphorylation of AGO2 may affect binding of miRNAs^12^. Thus, to check whether AGO2 tyrosine phosphorylation status was modified in GRB2 knockout cells, we immunoprecipitated AGO2 from serum starved G1 and HEK293T cells. Tyrosine phosphorylation could not be detected on AGO2 in either cell line (Extended Data Fig. 8), indicating that GRB2 did not affect tyrosine phosphorylation of AGO2 under these conditions.

Overall, these data indicate that knocking out of GRB2 regulated two groups of miRNAs by different mechanisms. First, production of the pro-oncogenic miR-17~92 cluster and mir-221 family of miRNAs was inhibited early in the biogenesis pathway, which may indicate a role for GRB2 in promoting transcription of these miRNAs. Second, expression of the tumour suppressor let-7 family of miRNAs was augmented via either stabilisation or enhanced loading of the mature miRNA. Considering the association of GRB2 with AGO2 and DICER1, we suggest that GRB2 may bind AGO2 to obstruct association of miRNAs during the RISC-loading step.

### let-7 and their targets are inversely dysregulated upon GRB2 knockout

Since the influence of GRB2 binding to AGO2 appears to directly affect the processing of members of the let-7 family miRNAs, we focused on investigating whether the presence of GRB2 has an impact on oncogenic protein expression. The let-7 family of miRNAs are well characterised tumour suppressors with expression which is frequently diminished in cancer^44^. Let-7 is known to target many oncogenes including the GTPase *RAS*, the let-7 regulator *LIN28B*, and transcription factors *HMGA1* and *HMGA2*, which have been shown to be dysregulated in lung, hepatocellular and intestinal cancer^46–49^. In the absence of GRB2 increased let-7 expression may result in enhanced activity and thus greater suppression of their targets. In support of this, qPCR analysis demonstrated that messenger RNA (mRNA) expression of five let-7 targets (*RAS*, *LIN28B*, *HMGA1*, *HMGA2, DICER1*) was reduced in serum-starved G1 cells (Fig. 5a)^50^. Western blotting demonstrated similar decreases in DICER1, LIN28B and HMGA2 protein levels (Fig. 5b, c). Thus, it was possible that the reduction in GRB2 correlated with an increase in AGO2-loaded let-7 which augmented let-7 silencing of target oncogenes.

To investigate if GRB2-mediated regulation of AGO2 could affect the cell cancer phenotype we performed migration assays in HEK293T cells which were overexpressing GFP-tagged AGO2 WT or the GRB2-binding-deficient mutant GFP-AGO2 4A (Fig. 2d). Cell migration was measured under conditions of reduced growth factor (1% FBS) for a period of 24 h. At all time points, inhibition of the interaction between GRB2 and AGO2 diminished cell migration (p < 0.05, Fig. 5d, Extended Data Fig. 9). Our data are therefore consistent with GRB2 playing a regulatory role in oncogene expression, whereby inhibition of GRB2 enhances let-7 suppression of oncogenes and thus represses migratory properties of the cell.

## Discussion

AGO2 function is dependent on manifold protein interactions^1^. Here we report a key addition to this group through demonstrating the direct interaction between GRB2 and AGO2, and the involvement of this complex in the regulation of miRNA expression. Mapping of the GRB2 interactome has previously revealed association with both miRNA biogenesis factors and mediators of gene silencing, suggesting GRB2 may play a larger role in miRNA-mediated translational repression than has previously been identified^51^. The herein described GRB2 interaction is the first to be reported for the AGO2 PAZ domain. Indeed, the only reported protein–protein interaction mediated by a PAZ domain in any protein is between DICER1 and Flock house virus B2 (FHVB2) protein and is not SH3 domain-mediated^52^. The PHLP motif is conserved in all four human AGO proteins, suggesting the potential for GRB2 to bind AGO1, 3 and 4 in a similar manner. GRB2-binding these other AGO proteins may also contribute to gene regulation described using total cell RNA extracts (Fig. 4b, 5a).

The potential for dimeric GRB2 to form a heterotetramer with two AGO2 molecules raises the possibility of GRB2 mediating the assembly of more than one RLC at elevated concentrations. Formation of higher order RISC structures is observed in miRNA-mediated gene silencing; cooperative targeting of a single mRNA by multiple AGO2-miRNA complexes results in enhanced silencing^53^ and can be mediated by TNRC6^54^. Additionally, AGO2-TNRC6 may form phase separated droplets with augmented deadenylation activity^55^.

Enhanced expression and activity of mature let-7 family miRNAs in GRB2 knockout cells (Figs. 4d, 5a) suggests that GRB2 inhibits their loading or promotes their turnover. However, it is important to note that further experiments are required to determine how GRB2 regulates these miRNAs and which stage of miRNA biogenesis is affected. Since we observe that GRB2 is able to form a complex with AGO2 and DICER1 (Fig. 3a-d), we propose that the presence of GRB2 inhibits miRNA loading. Considering the proximity of ^323^PHLP^326^ to the docking site for the miRNA 3’ terminus (Fig. 3f), and as GRB2 does not preclude AGO2 binding DICER1 (Fig. 3b-d), direct interaction of GRB2 with AGO2 potentially induces a conformational change in the AGO2 PAZ domain which stalls loading of let-7 miRNAs. Structural changes are central to the miRNA-loading process; for example, prior to miRNA binding AGO2 adopts multiple closed conformations then the HSC70/HSP90 chaperones bind and open AGO2, allowing it to accommodate miRNA duplexes^56^. While our MST data indicate formation of a trimeric complex containing GRB2, AGO2 and DICER1 (Fig. 3b-d), additional data is needed to elucidate how this structure is formed and if it exists in the context of the cell.

An important question is why the loading of only some miRNAs was upregulated upon GRB2 depletion. Comparisons can be drawn with the association of TP53 mutants with AGO2 which was previously suggested to regulate association specifically with let-7 miRNAs through changes in conformation^57^. Alternatively, preference of DICER1 for long-loop pre-miRNAs was proposed to mediate suppression of loading these miRNAs via AGO2 Y393 phosphorylation^18,58^, suggesting that the reliance for DICER1-mediated loading may select miRNAs with specific features. Multiple other structural and sequence characteristics of pre-miRNA have been shown to influence DICER1 efficiency and specificity of processing and loading miRNA^59,60^, but it remains to be determined if let-7 possess features such as these.

GRB2 control of let-7 appears to mediate translational de-repression of oncogenes including RAS (Fig. 5a) suggesting that GRB2 may have dual roles in promoting the MAPK pathway; i.e. through both its canonical function as an adaptor protein linking activated receptors to Sos/RAS, and via regulation of miRNA expression and hence RAS protein concentration. As the GRB2-AGO2 interaction is SH3-mediated, complex formation is solely dependent on GRB2 concentration, and hence fluctuating intracellular GRB2 expression may surpass a threshold whereby the GRB2-AGO2 complex dominates and MAPK signalling is enhanced. Thus, strict control of GRB2 expression is required to abrogate aberrant oncogene expression and cell proliferation. While genetic amplification of GRB2 is not a feature of tumour cells, enhanced GRB2 expression has been recorded in various cancers^61–63^. Furthermore, the relatively tight affinity measured for GRB2 with AGO2 suggests complex formation may occur even when the GRB2 concentration is low (Fig. 2a).

Consistent with the augmentation of tumour suppressor let-7 miRNA, GRB2 knockout inhibited expression of pro-oncogenic miRNA families mir-17, mir-19 and mir-221 (Fig. 4c). Many targets of these miRNAs are tumour suppressor genes, such as the phosphatase PTEN which negatively regulates AKT signalling^64,65^. Control of these miRNAs is likely to be transcriptional as several members of the same cluster are affected. Transcription of both the mir-19~92 and mir-221 clusters is activated by nuclear factor κB (NF-κB) and STAT3^66–68^. Both transcription factors are activated by growth factor-initiated signalling pathways, making it difficult to postulate the regulatory mechanism at play under the non-stimulated conditions adopted in this study. However, considering the involvement of GRB2 in these pathways^69,70^, it is possible that altered GRB2 expression could disrupt the mechanisms that regulate these transcription factors.

In summary, we present evidence for a regulatory role of GRB2 in the biogenesis of two groups of miRNAs. First, GRB2 augments expression of the mir-17~92 and mir-221 clusters, which we hypothesise is via transcriptional activation. Second, GRB2 forms a direct interaction with AGO2 and consequently associates with DICER1; this complex may be responsible for GRB2-mediated inhibition of let-7 loading. Thus, GRB2 can regulate oncogene expression and may have consequences for tumour pathogenesis.

## Methods

### Cell culture

HEK293T, PC3 and NIH3T3 cells were cultured in DMEM (Gibco, 11520416), while A498 cells were cultured in MEM (Gibco, 11095080). All cell lines were cultured in media containing 10% foetal bovine serum (FBS, Sigma-Aldrich, F7524), in 5% CO_2_ and at 37 °C.

GRB2 knockout was achieved using the homology-directed repair (HDR)-mediated knockout kit (OriGene, KN200469), which utilises CRISPR/Cas9 technology to insert puromycin resistance and GFP genes into the start of *GRB2*. *GRB2* alleles may therefore be knocked out by HDR or non-homologous end joining, which can introduce insertions or deletions into the gene. Clones with complete or partial GRB2 knockout were validated by genotyping and western blotting. Genotyping confirmed that clone 2 expressed one *GRB2* allele with a 20 nt deletion (aligning to aa1-8 in the NSH3 domain), as well as a large 5’ insert from the CRISPR donor DNA, aligning to GFP (Extended Data Fig. 10). The NSH3 deletion disrupts the beta-barrel structure of this domain making it unlikely that it folds correctly. The translated protein therefore contains a fragment of the GFP protein fused to the N-terminus GRB2 aa9-217, producing a protein of molecular weight ~ 30 kDa (Fig. 4a).

### Plasmids

For biophysical experiments, AGO2 domains (N-terminal, residues 1–139; PAZ, residues 235–348; MID, residues 450–573; PIWI, residues 517–818) were inserted into the MBP-fusion vector pMAL-c5x, producing proteins with N-terminal MBP-tags. AGO2 PAZ domain (residues 228–348) was inserted into pET28a to produce PAZ with an N-terminal His-tag. The PrimeSTAR GXL polymerase kit (Takara Bio, R050) was used to mutate of AGO2 ^323^PHLP^326^ to a stretch of four alanine residues in pMAL-c5x-PAZ. GRB2 W36K, W193K and R86A point mutations were made in pcDNA-3.1-RFP-N-GRB2 (GRB2 with N-terminal RFP fusion) using Vent polymerase followed by Dpn1 digestion.

### Western blotting

Cells were lysed in lysis buffer (50 mM Hepes–NaOH pH 7.5, 1% [v/v] Igepal-CA630 [Sigma, 18896] 10 mM NaF, 1 mM Na_3_VO_4_, 10% [v/v] glycerol, and 150 mM NaCl) containing 1 × Calbiochem protease inhibitor (PI) cocktail III (Fisher Scientific, 12818395). Following quantification by Bradford assay (Thermo Scientific, 23238), equal amounts of protein were loaded onto a 4–20% polyacrylamide precast gel (Bio-Rad, 4561094) and separated by SDS-PAGE. Proteins were transferred to a PVDF membrane using the iBlot 2 Gel Transfer Device (Invitrogen, IB21001) and transfer stacks (Invitrogen, IB24001). Membranes were blocked in TBST wash buffer (50 mM Tris–HCl pH 7.6, 150 mM NaCl, 1 mM EDTA, and 0.1% [v/v] Tween-20) containing 3% (w/v) BSA, for a minimum of 30 min. before addition of primary antibodies. Primary antibody incubation was overnight at 4 °C, followed by six 10 min washes in TBST buffer at room temperature. Blots were incubated with HRP-conjugated secondary antibody in TBST buffer containing 3% BSA, for 1 h at room temperature, and washed as before. Membranes were imaged using western ECL substrates (Bio-Rad, 1705061) and visualised on a G:Box Chemi XT4 (Syngene). If bands were faint, strong western blotting substrate (Thermo Scientific, 34094) was used as a 10 × dilution in western ECL substrates. To detect high levels of protein, PVDF membranes were stained with ponceau, prior to blocking in BSA. Membranes were incubated with ponceau (Sigma-Aldrich, P3504) for 10 min at room temperature, before washing in distilled water until background stain was removed. Band intensity was quantified using ImageJ and normalised to that of the loading control GAPDH or α-Tubulin.

Intact blots are shown in Extended Data Fig. 12.

### Immunoprecipitation

Prior to lysis (see western blotting) cells were starved by washing three times in phosphate-buffered saline (PBS) and overnight incubation in media without FBS. For experiments to analyse protein pulldown, reactions were made using 500 µg protein in 500 µl and cleared by incubation with Protein A/G magnetic beads (Pierce, 88802) which had been conjugated with control IgG antibody for 4 h at 4 °C. Lysates were separated from beads using a magnetic separator and incubated with 4 µg antibody overnight at 4 °C, before addition of magnetic beads for a further 4 h. Beads were washed three times in 1 ml lysis buffer and bound protein analysed by western blotting.

Where immunoprecipitates were to be used for RNA quantification, lysates were made using lysis buffer (25 mM Tris–HCl pH 7.4, 150 mM KCl, 5 mM EDTA, 0.5 mM DTT, 0.5% NP-40, 100 U/ml RNase inhibitor (New England Biolabs (NEB), M0314), and 1 × Calbiochem PI cocktail III). 100 µl lysate was made to 1 ml using immunoprecipitation buffer (10 mM Tris–HCl pH 7.5, 150 mM NaCl, 0.5 mM EDTA, and 100 U/ml RNase inhibitor) and incubated overnight at 4 °C with Magna ChIP protein A/G magnetic beads (Sigma-Aldrich, 16–663), which had previously been conjugated with 5 ug antibody. Beads were washed six times with 1 ml wash buffer (20 mM Tris–HCl pH 7.5, 150 mM NaCl, 0.4% NP-40 (v/v), and 100 U/ml RNase inhibitor), before RNA was extracted.

### Fluorescence resonance energy transfer, FRET

Cells were seeded onto coverslips and co-transfected with 1 µg each GFP and RFP-tag plasmids using Metafectene (Biontex, T020) at 50% confluency. Proteins were expressed for 48 h and cells starved overnight (see Immunoprecipitation) before cells were fixed by incubation in 4% (w/vol) paraformaldehyde (PFA) (Boster Biological Technology, AR1068) for 20 min at room temperature. Coverslips were attached to slides using mounting media (Sigma-Aldrich, F6057). Microscopy was done using a LSM880 + airyscan upright (Zeiss).

### Fluorescence lifetime imaging microscopy, FLIM

HEK293T cells with stable FGFR2 expression were transfected with designated plasmids. 24 h after transfection cells were detached and seeded onto coverslips and allowed to grow for a further 24 h before serum starvation (see Immunoprecipitation). Cells were fixed by addition of 4% (w/vol) PFA, pH 8.0, washed six times and mounted onto a slide with mounting medium (0.1% p-phenylenediamine and 75% glycerol in PBS at pH 7.5–8.0). FLIM images were captured using a Leica SP5 II confocal microscope with internal FLIM detector. GFP was excited at 900 nm with titanium–sapphire pumped laser (Mai Tai BB, Spectral Physics) with 710–920 nm tunability and 70 femtosecond pulse width. Becker & Hickl (B&H) SPC830 data and image acquisition card was used for time-correlated single photon counting (TCSPC); electrical time resolution was 8 picoseconds with a pixel resolution of 256 × 256. Data processing and analysis were performed using B&H SPC FLIM analysis software. The fluorescence decays were fitted to a single exponential decay model.

### Migration assay

Cells were seeded in the Radius™ 96-Well Cell Migration Assay plate (Cell Biolabs, CBA-126) and transfected with 100 ng GFP plasmid using Metafectene. Proteins were expressed for 24 h and transferred to DMEM containing reduced growth factor (1% FBS) 16 h before the migration assay was started. Phase contrast images were captured every 4 h for a total period of 24 h using the IncuCyte S3 (Sartorius) and the area migrated over was quantified using ImageJ.

### Quantitative polymerase chain reaction, qPCR

RNA was extracted from HEK293T or G1 cells (G1 cell data derived from both G1.1 and G1.2 and combined) using TRIzol reagent (Invitrogen, 15596026) and contaminating DNA removed using the DNA-free DNA removal kit (Invitrogen, AM1906). cDNA was synthesised using the miScript II reverse transcriptase kit (Qiagen, 218161). miRNA, precursor miRNA and mRNA expression changes were measured by qPCR using the miScript SYBR green PCR kit (Qiagen, 218073) and the Rotor-Gene-Q cycler (Qiagen). When total cellular RNA was extracted, precursor and mature miRNAs were normalised to the small nuclear RNA RNU6-6P, whereas the housekeeping gene GAPDH was used for mRNAs. Fold change was calculated using the ΔΔct method.

In experiments analysing AGO2-bound RNA, RNA extraction and qPCR was completed as above, with the following changes. Prior to RNA extraction using TRIzol both input samples and immunoprecipiates were incubated for 30 min at 55 °C with proteinase K to digest protein. Ct values were used to calculate the fold enrichment of RNA in immunoprecipitates over input, followed by normalisation to GAPDH and of G1 to HEK293T.

### Small RNA sequencing

Total cell RNA was extracted and DNase-treated (see qPCR). Library preparation and sequencing were done using the NEXTFLEX small RNA-seq kit (Perkin Elmer, NOVA-5132). Sequencing was performed on the HiSeq 1500 sequencer (Illumina). Adaptor sequences and random bases were trimmed using Cutadapt^71^ and read quality assessed with FastQC (bioinformatics.babraham.ac.uk/projects/fastqc). Sequencing reads were aligned to the human genome assembly GRCh38 (GENCODE, Release 31)^72^ using Bowtie^73^. A count matrix was produced using featureCounts^74^ which was based on the microRNA annotation file from miRbase (v22)^75^, before differential expression was analysed using the DEseq2 package^76^. miRNAs were selected if they achieved statistical significance at p < 0.05. Two independent biological replicates were performed.

### Bacterial protein expression and purification

All proteins for GST-pulldown and biophysical experiments were expressed in BL21(DE3) cells (NEB, C2527). Bacterial cells were lysed by sonication (Fisherbrand Model 120 Sonic Dismembrator, Fisher Scientific, 12337338). Following purification (see GST-pulldown, ITC, MST), protein purity was analysed by SDS-PAGE and Coomassie staining.

### GST-pulldown

GST-tagged proteins were extracted in wash buffer A (20 mM Tris–HCl pH 8, 150 mM NaCl, and 1 mM β-mercaptoethanol) by incubation with glutathione superflow resin (Clontech, 635608), followed by washing until no protein could be detected by Bradford assay. The amount of bound protein was analysed by SDS-PAGE and staining with Coomassie dye. Volumes of bead-immobilised proteins were adjusted to ensure equal volumes of protein and made equal with unbound glutathione superflow resin, then incubated with 1 mg cell lysate in 500 µl for 1 h at room temperature. Beads were washed as for immunoprecipitation experiments and bound proteins analysed by western blotting.

### Isothermal titration calorimetry, ITC

MBP-tagged AGO2 domains were used in ITC with GRB2. For the N-terminal domain of AGO2, a construct was used containing residues 1–139, to test for binding of GRB2 to PRMs in the N-terminal region adjacent to this domain.

MBP-tagged proteins were extracted in wash buffer B (20 mM Tris–HCl pH 8, 150 mM NaCl, 10% (v/v) glycerol, and 1 mM β-mercaptoethanol) using a 5 ml MBPTrap HP column (GE Healthcare, 28918778). Non-specifically bound protein was removed by washing with 1 L and MBP-proteins were eluted by application of wash buffer B containing 10 mM maltose. Proteins were purified further by size exclusion chromatography (SEC) using a Sephadex G-200 column (271233, Sigma Aldrich).

His-GRB2 was purified in wash buffer A by incubation of cell lysates with TALON metal-affinity resin (Takara Bio, 635504). The resin was washed as for GST purification (see GST-pulldown) and His-GRB2 was eluted in wash buffer A containing 200 mM imidazole.

Prior to ITC, all proteins were dialysed and peptides dissolved into matched buffer. Wash buffer B was used for experiments using MBP-tagged AGO2 domains and wash buffer A for peptides. The concentration of dissolved peptides was estimated using absorbance at A280 due to presence of a single tyrosine residue in each peptide.

500 μM MBP-tagged AGO2 domain was titrated into 20 μM His-tagged GRB2. An initial 0.5 μl injection was followed by 19 2 μl injections, spaced every 120 s. For experiments using peptides, 1.2 mM peptide was titrated in a solution of 80 μM His-tagged GRB2, with an initial injection of 0.5 μl followed by 13 3 μl injections and spacing of 90 s. All experiments were completed at 25 °C on a Microcal iTC200 (Malvern Analytical)). Excluding the initial peak, peaks were integrated to give a Wiseman curve, which was fitted with Microcal Origin to yield the binding affinity, *K*_D_, and stoichiometry, n. Binding curves were symmetrical and fit with a one site of binding model, indicating that binding was 1:1. However, the measured stoichiometry was > 1, suggesting that RNA remaining in the protein preparation oligomerised PAZ and resulted in binding of multiple PAZ domains to a single site on GRB2. Therefore, the curve was fit with n fixed at 1 to calculate *K*_D_.

### Microscale thermophoresis, MST

To confirm that the presence of RNA in the PAZ preparation used in ITC did not affect the measured affinity, MST of GRB2 and PAZ was completed using PAZ which had and had not been treated with RNase H (Extended Data Fig. 11a, b). His-tagged GRB2 and AGO2 PAZ domain were purified (see ITC) and, where stated, contaminating RNA was removed from PAZ by incubation of cell lysates with 20 μg/ml RNase H for 16 h at 4 °C. Prior to MST, all proteins were dialysed into MST buffer A (20 mM HEPES–NaOH pH 7.5, 150 mM NaCl, 10% glycerol, and 1 mM β-mercaptoethanol). Protein was labelled by incubation of 100 µM protein with 200 µM Atto 488 NHS ester (Sigma-Aldrich, 41698) for 1 h. Labelled protein was separated from unconjugated dye on a PD10 desalting column (GE, 17–0851-01). A two-fold dilution series of unlabelled PAZ was set up and mixed with labelled GRB2, yielding final concentrations of 100 nM GRB2 and 20–653,000 nM PAZ. Proteins were transferred to capillaries (Monolith, MO-AK005) and fluorescence was recorded using a Monolith, NT.115. Atto 488 was excited with a blue LED set to a power level that achieved fluorescence between 800 and 1200 count. MST power was used at 20% and reactions were performed in triplicate.

For experiments using DICER1, the DICER1^CT^ construct (residues 1276–1922) was purified using wash buffer C (20 mM Tris–HCl pH 8, 300 mM NaCl, and 1 mM β-mercaptoethanol). Lysates were first incubated with RNase to remove RNA, then TALON metal-affinity resin, followed by washing in wash buffer C containing 20 mM imidazole and elution with 150 mM imidazole. GST-AGO2 was purified in wash buffer C in the presence of protease inhibitors. Lysates were incubated with RNase and GST-AGO2 extracted by incubation with glutathione superflow resin, then washed with 20 column volumes and eluted with 20 mM reduced glutathione. Protein was further purified by gel filtration using a Superdex 75 column (Cytiva, 17517401) and buffer exchanged into MST buffer B (20 mM HEPES, 150 mM NaCl and 1 mM TCEP pH 7.5). GST was cleaved by overnight incubation with thrombin (1 U per 1 mg protein), then thrombin and GST proteins were removed by incubation with Benzamidine Sepharose 4 Fast Flow beads (Cytiva, 17512310) and glutathione superflow resin respectively. GRB2 and DICER1^CT^ were dialysed into MST buffer B, before proteins were labelled and MST was performed as for GRB2 with PAZ. A dilution series of 3.66 to 120,000 nM unlabelled GRB2 and 100 nM labelled DICER1^CT^, or 2.29 to 75,000 nM unlabelled DICER1^CT^ and 100 nM labelled AGO2, was used.

### Circular dichroism, CD

CD studies were completed using 200 μl 0.2 mg/ml protein in a 1 mm path-length curvette, on a Chirascan CD spectropolarimeter (Applied Photophysics). Far ultraviolet (UV) absorbance was measured over wavelengths 190–260 nm.

### In silico docking

Prior to docking, structures of GRB2 (PDB: 1GRI^77^) and AGO2 PAZ domain (PDB: 6RA4^78^) were prepared and energy minimised using MDWeb^79^. Molecular docking was performed using HADDOCK2.2^40^. HADDOCK uses an information-driven approach to determine the structure of the protein complex and consistently performs well in Critical Assessment of PRedicted Interactions (CAPRI) experiments^80,81^. To facilitate the SH3-PxxP-mediated complex, the ^323^PHLP^326^ motif on AGO2 PAZ and residues in GRB2 NSH3 (Y7, F9, W36, F47, P49, and Y52, which define the hydrophobic binding pocket^41^), were selected as the 'active residues (directly involved in binding)'. Passive residues around the active residues were automatically included in the docking process. Both defined structures were prepared using PyMOL (Schrӧdinger, LLC).

### Statistics

Significance of differences were calculated using a two-tailed Student’s t-test. During DESeq2 analysis of miRNA expression, p values were adjusted using the Benjamini–Hochberg method to account for multiple significance testing^76^. For qPCR experiments, all statistics were performed on log2 transformed fold changes.

## Supplementary Information

Supplementary Information.

## Data Availability

The datasets generated during the current study are available from the corresponding author on reasonable request and will be available in the Sequence Read Archive repository BioSample accessions: SAMN35447638, SAMN35447639, SAMN35447640, SAMN35447641, SAMN35447642, SAMN35447643 with Temporary Submission ID: SUB13477181. Release date: 27th May 2025.
