# Supplementary material for: Regulation of microRNA expression by the adaptor protein GRB2
Source: Sci Rep. 2023 Jun 16;13:9784. doi: 10.1038/s41598-023-36996-3 (PMC10276003; doi:10.1038/s41598-023-36996-3)
Supplement: Supplementary file 1 — Supplementary Information. [file 41598_2023_36996_MOESM1_ESM.docx]

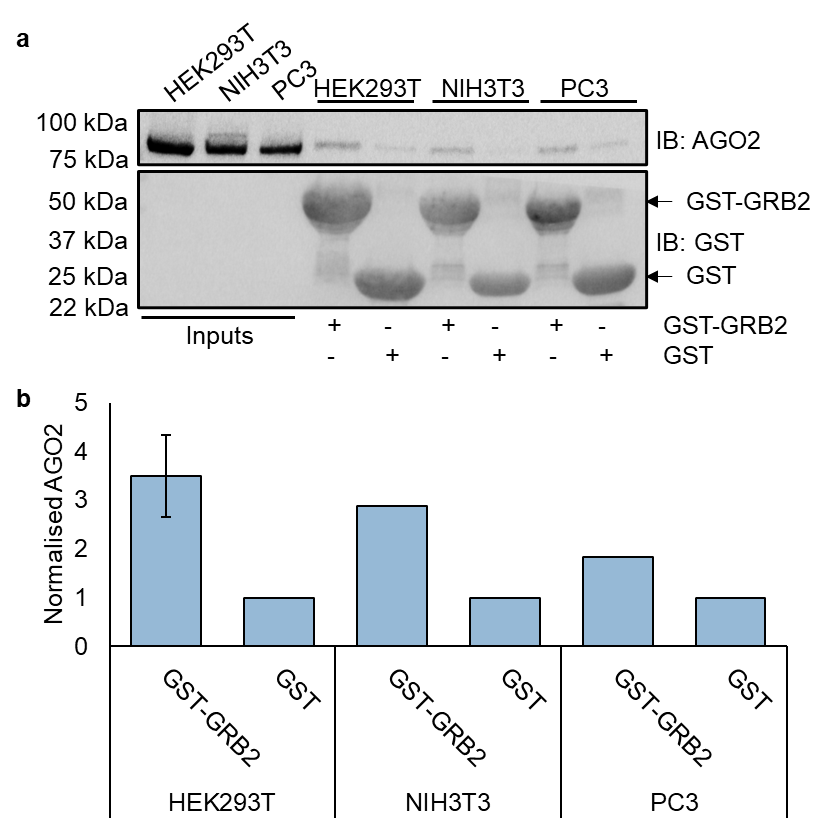


1. GRB2 complexes with AGO2 under non-stimulated conditions

**a,** Western blot of AGO2 recruitment to GST-tagged GRB2 in three cell lines which were deprived of growth factor. GST proteins were purified and immobilised on beads before incubation with cell lysates. A longer exposure was used to capture AGO2 bands than for GST bands. Both images are taken from the same western blot. **b,** Densitometry analysis of **a,** with AGO2 normalised against GST. N = 3 (HEK293T); N = 1 (NIH3T3, PC3).
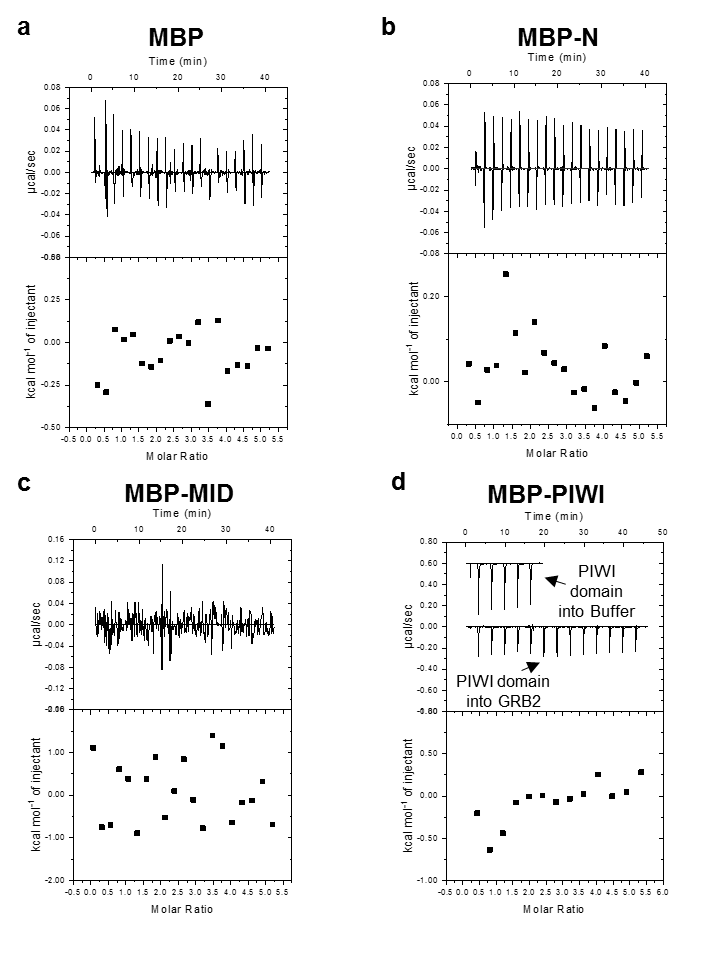


1. N-terminal, MID and PIWI domains of AGO2 do not bind GRB2

ITC of MBP alone or MBP-tagged AGO2 domains titrated into GRB2. The N-terminal domain construct contained residues 1-139 to additionally test for binding of proline rich motifs in the N-terminal adjacent region. A control titration of MBP-PIWI into buffer was also performed. Binding was not observed for MBP nor any of the domains.


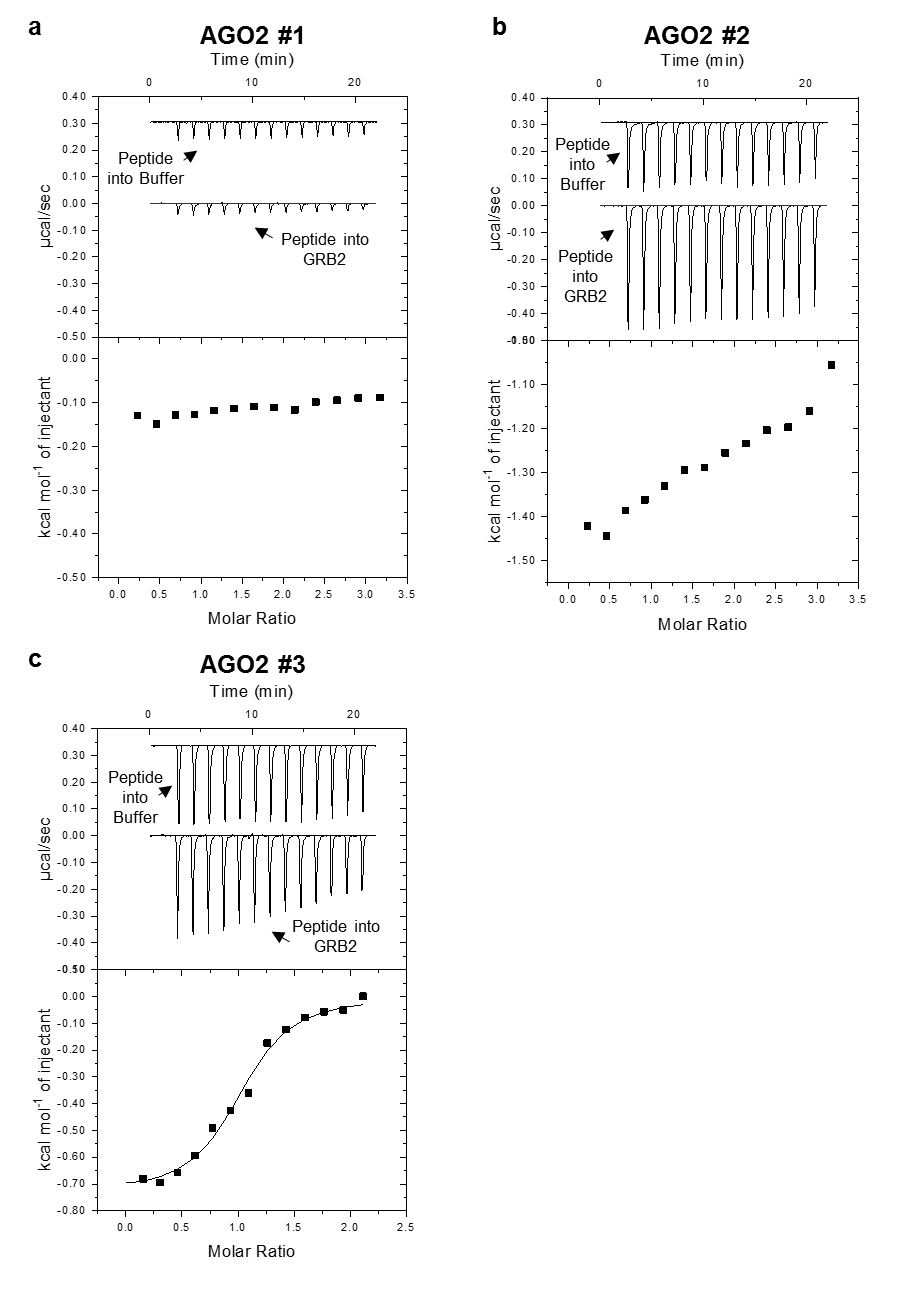


1. Proline-rich regions in AGO2 N-terminal domain do not bind GRB2

**a-b,** ITC of GRB2 with two peptides spanning each proline-rich motif in AGO2 N-terminal domain. Peptides were titrated into GRB2 or buffer as a control. Binding was not observed.


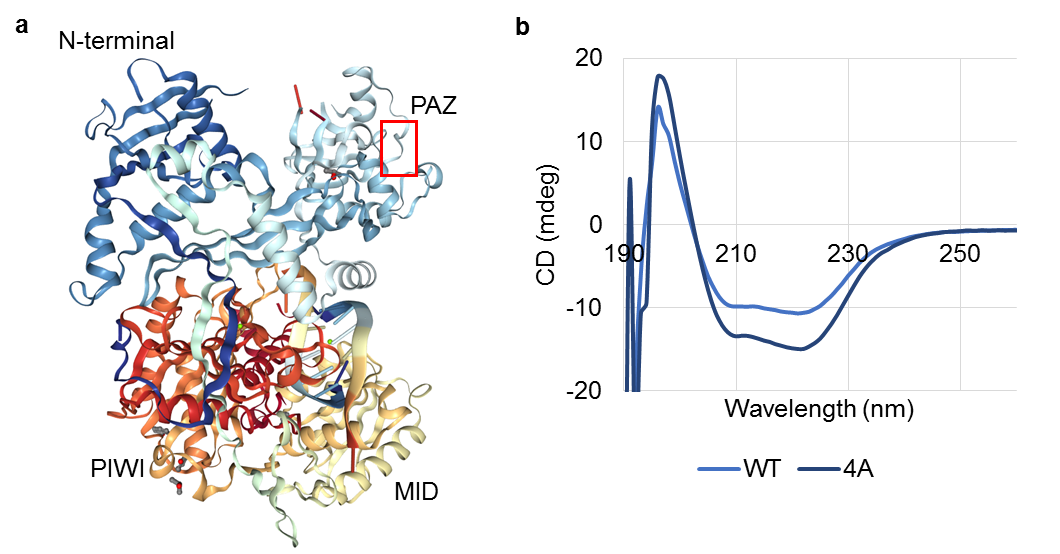


1. Mutation of ^323^PHLP^326^ does not result in PAZ unfolding

**a,** Crystal structure of AGO2 containing ^323^PHLP^326^ (inside red box). (PDB: 4W5N^1^). Figure generated using PyMOL. **b,** Far-UV circular dichroism spectra of wild type (WT) and ^323^PHLP^326^-mutated (4A) MBP-PAZ secondary structure. WT and 4A mutant PAZ domain have highly similar structures. They contain ~50% α-helix content (53.8% vs 48.4% respectively), negligible β-sheet content (0% vs 0.1% respectively) with similar content of loops/unstructured regions (46.2% vs 51.5% respectively)


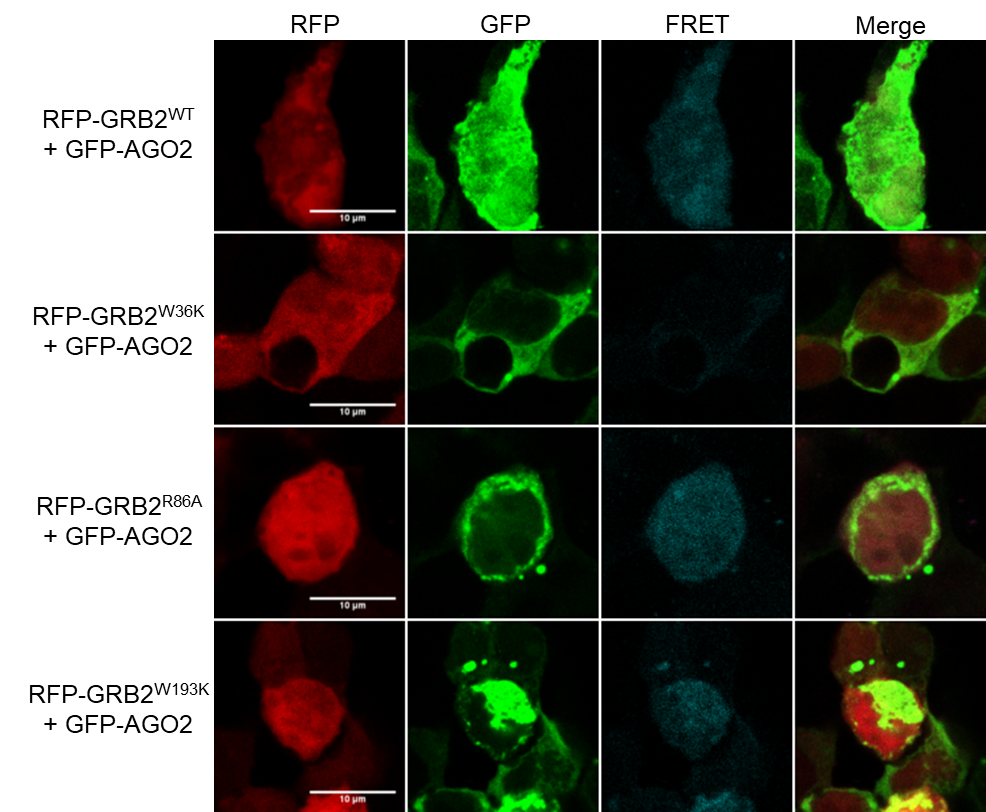


1. Mutation of GRB2 NSH3 abrogates binding to AGO2

Fluorescence resonance energy transfer (FRET) between WT and mutant RFP-tagged GRB2 and GFP-tagged AGO2 proteins expressed in serum-starved HEK293T cells. The R86A mutant has lost SH2 binding capacity, whereas the W36K and W193K mutants have lost the binding function of the NSH3 and CSH2 domains respectively. N = 3. Scale bars are 10 μm.


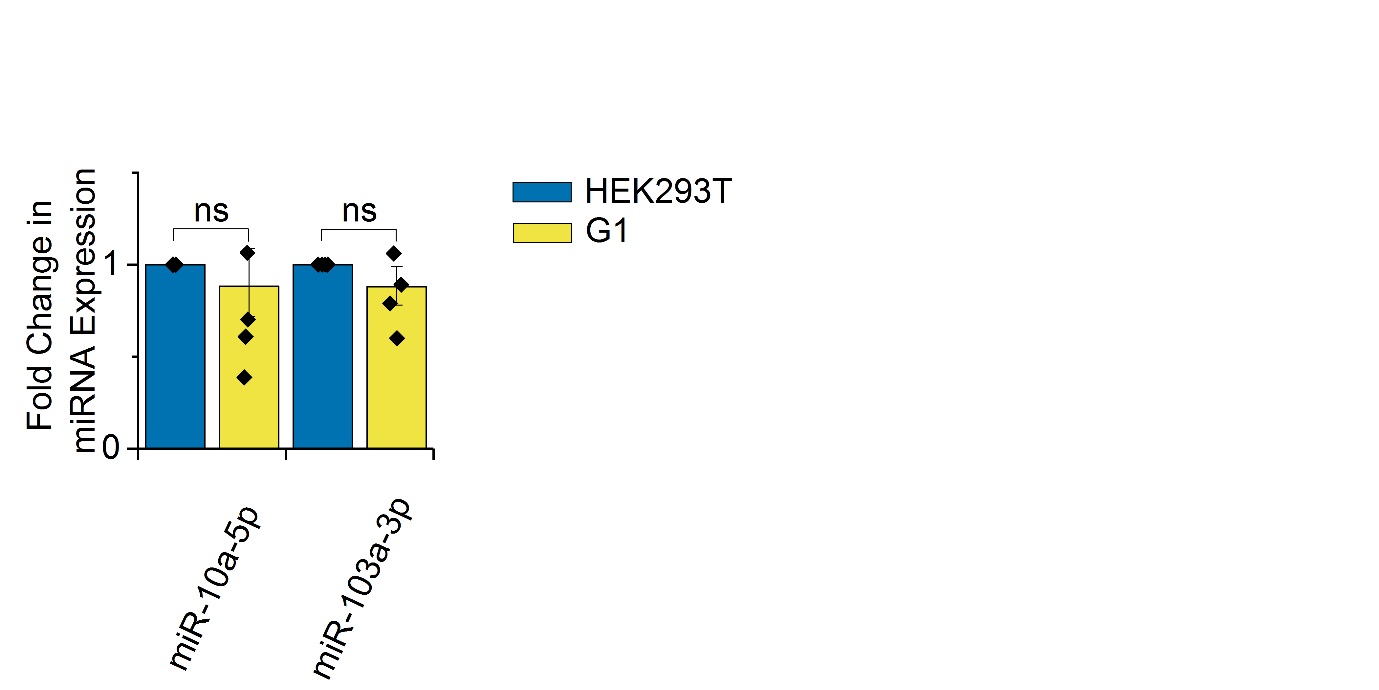


1. Control miRNAs are not differentially regulated in G1

Control miRNAs, which were found to have similar expression levels in HEK293T and G1 by RNA sequencing, are also expressed at equal levels when measured by qPCR. Total cell RNA was extracted from serum-starved HEK293T and G1 cells and cDNA quantified by qPCR, using the housekeeping gene RNU5G to normalise expression. Comparisons were made using a two-tailed Student’s t-test and error bars show standard error of mean. N = 4. ns = not significant.


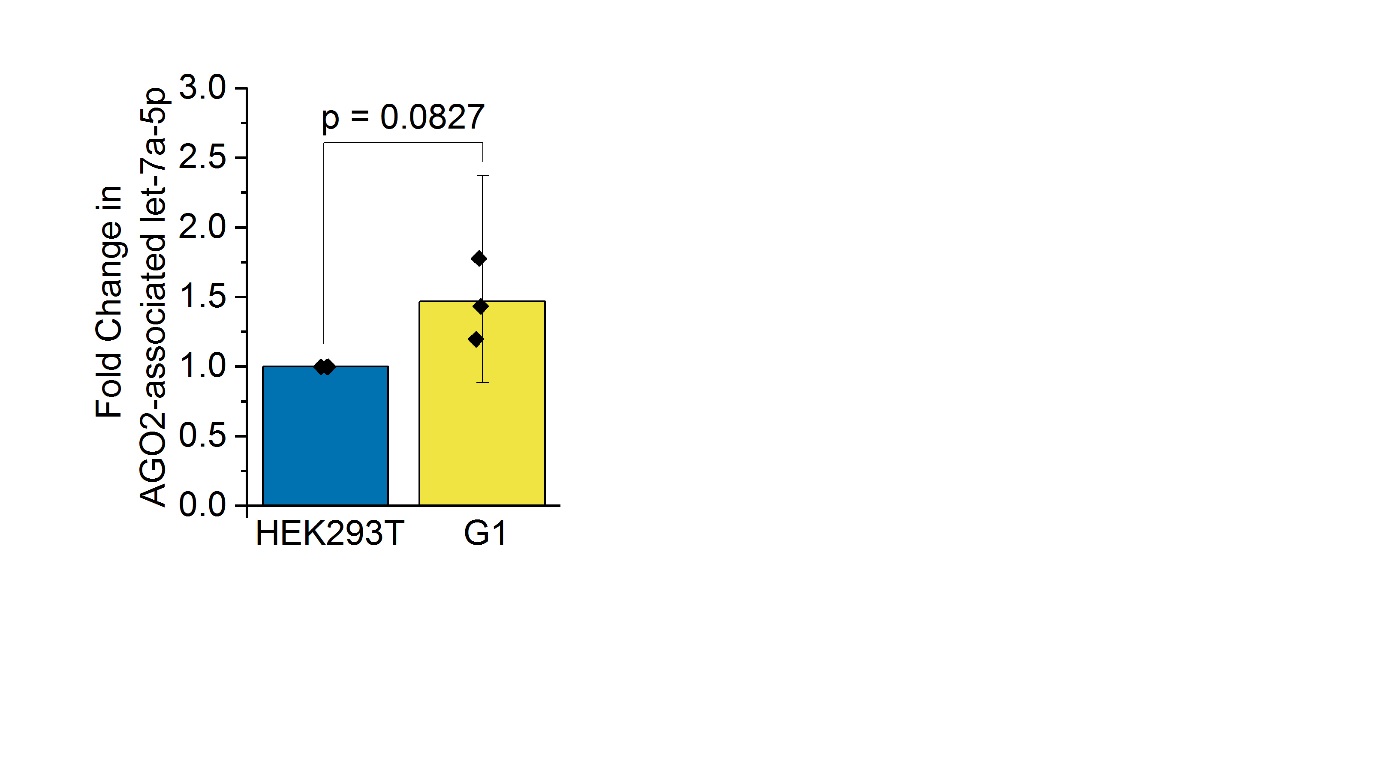


1. Binding of let-7a-5p to AGO2 is enhanced in G1

AGO2 was immunoprecipitated from HEK293T and G1 cells under conditions of growth factor deprivation. AGO2-bound miRNA was measured by qPCR. Comparisons were made using a two-tailed Student’s t-test and error bars show standard error of mean. N = 3.


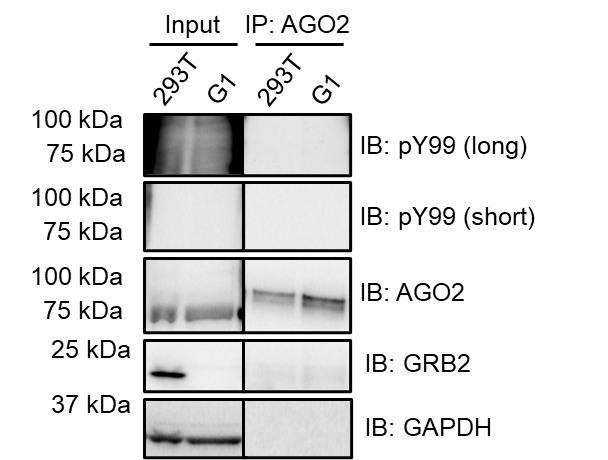


1. AGO2 is not tyrosine phosphorylated in serum starved HEK293T cells with or without GRB2 knockout

AGO2 was immunoprecipitated (IP) from serum starved HEK293T (293T) and G1 cells. Tyrosine phosphorylation was assessed by western blotting with the pY99 antibody. Images captured with both long and short exposures are shown for pY99, whereas only the image captured with a long exposure is shown for AGO2. Short exposures were used to capture GRB2 and GAPDH bands. All images are taken from the same western blot.


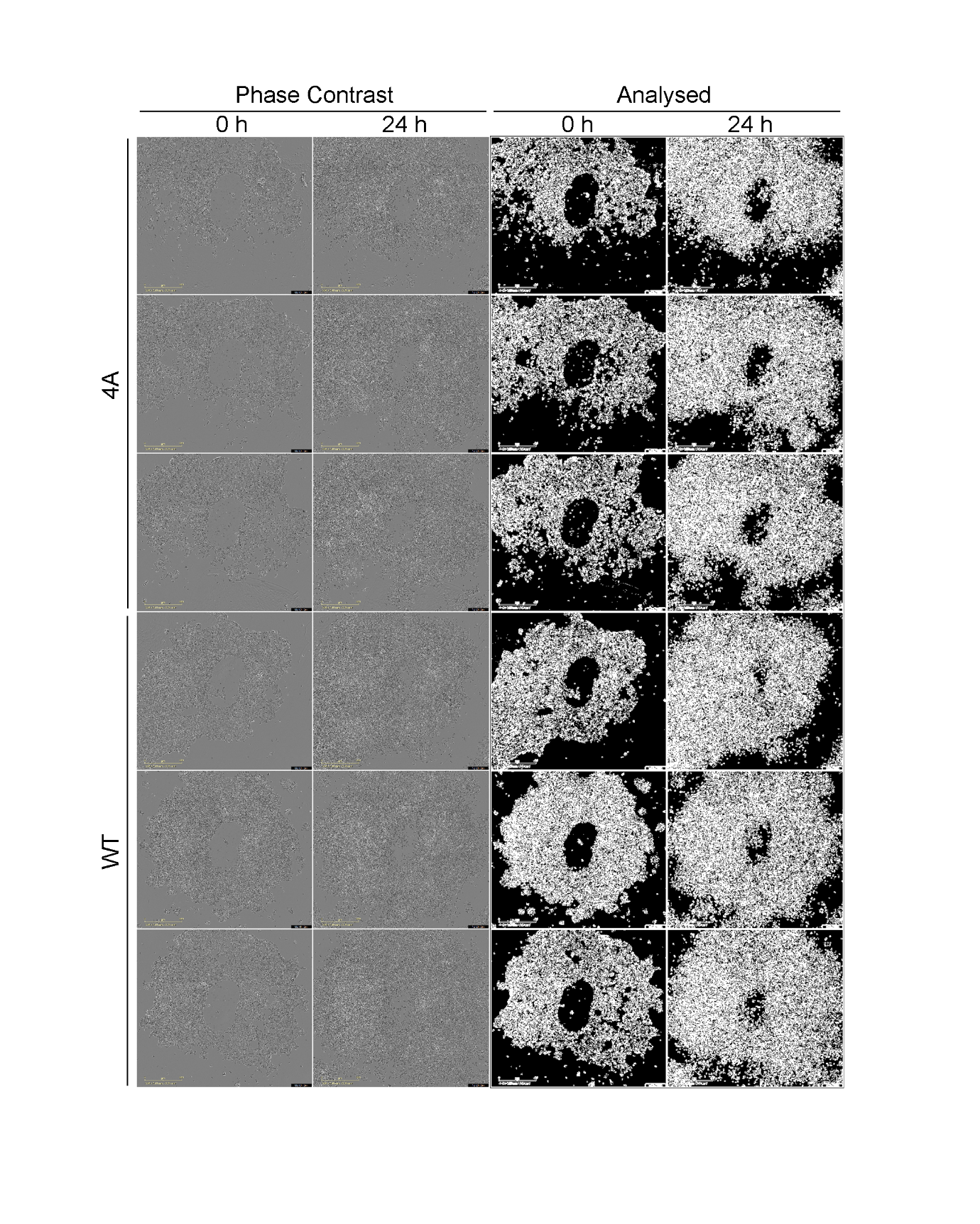


1. Inhibition of GRB2 regulating AGO2 diminishes cell migration

HEK293T cells overexpressing wild type (WT) GFP-tagged AGO2 or AGO2 which is deficient in GRB2-binding (4A) were cultured in growth-factor-reduced media (1% FBS). Cell migration into a central cell-free area was monitored by capturing phase contrast images every four hours (h) for a total of 24 h. Images were analysed using ImageJ. N = 3.


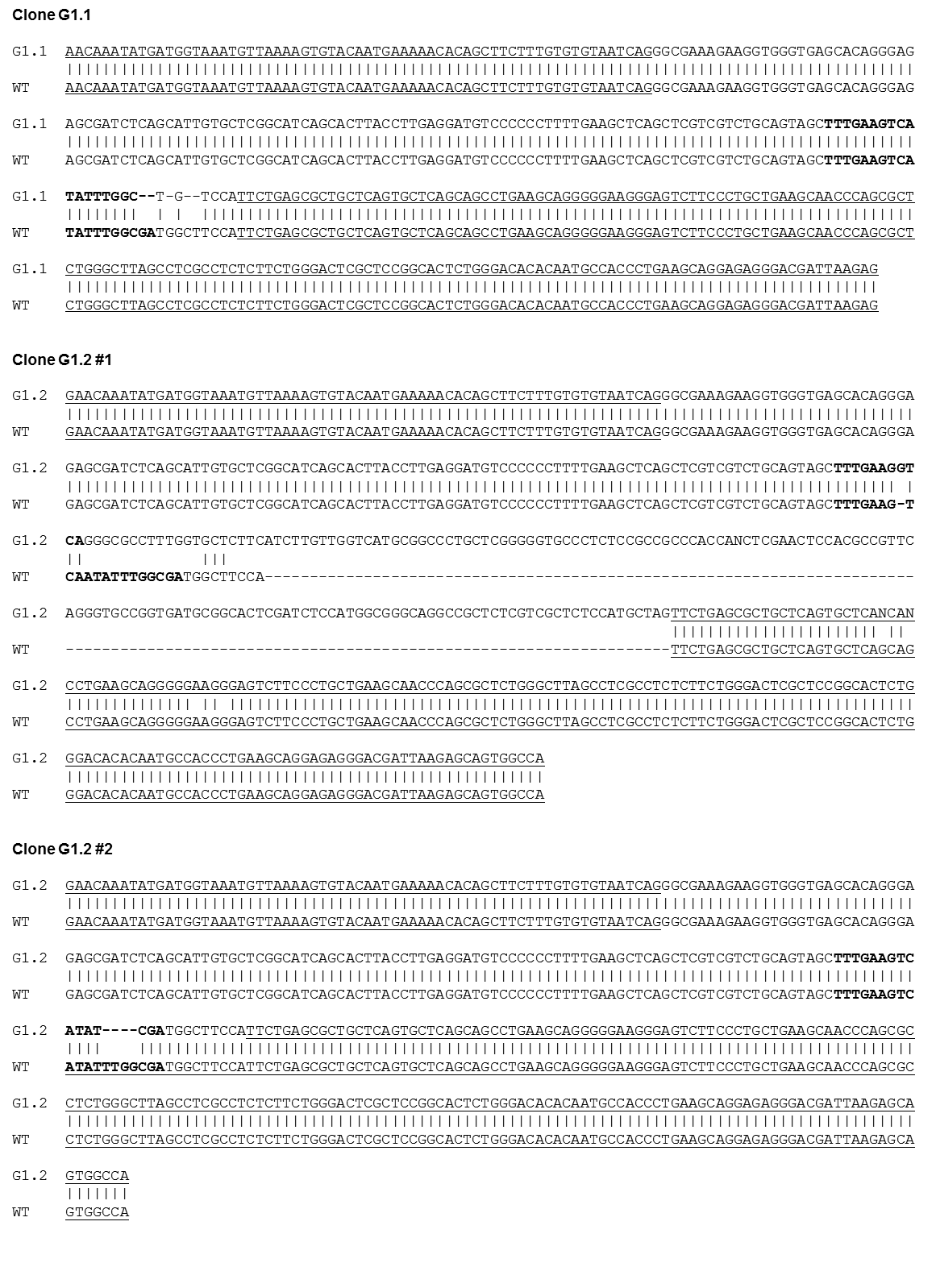


1. Alignments of mutated *GRB2* alleles in G1 with WT *GRB2*

Genomic sequencing of GRB2-depleted HEK293T clones (G1) confirmed mutations in *GRB2* alleles. G1.1 expressed one allele with a 5 nt deletion leading to complete GRB2 knockout. G1.2 expressed one allele (#1) with a 20 nt deletion and 140 nt insertion and one allele (#2) with a 4 nt deletion. As both clones survived under puromycin treatment, they also contained one GRB2 allele which had been knocked out by homology directed repair (HDR), which inserted GFP and puromycin resistance genes at the start of *GRB2*. Thus, G1.1 had complete GRB2 knockout whereas G1.2 expressed an N-terminally mutated GRB2 protein. Sequences aligning to homologous arms used for HDR are underlined and the sequence targeted by the guide RNA used to direct Cas9-mediated cutting is in bold. Alignments were made using Blast^2^.


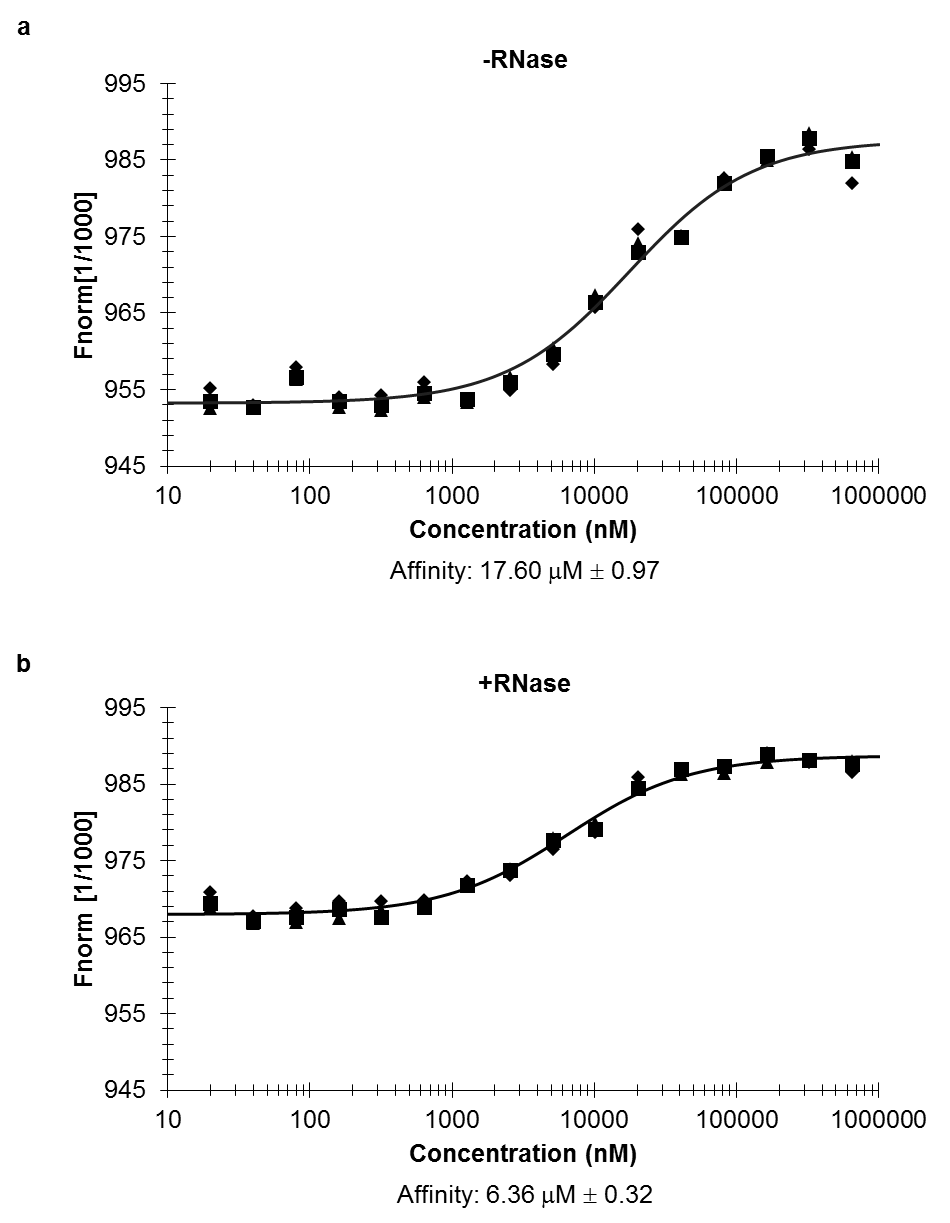


1. GRB2 binds AGO2-PAZ regardless of RNA-association

**a, b,** MST of GRB2 with PAZ domain of AGO2 which has or has not been treated with RNase. Similar binding affinities were observed for PAZ in the presence and absence of RNA.


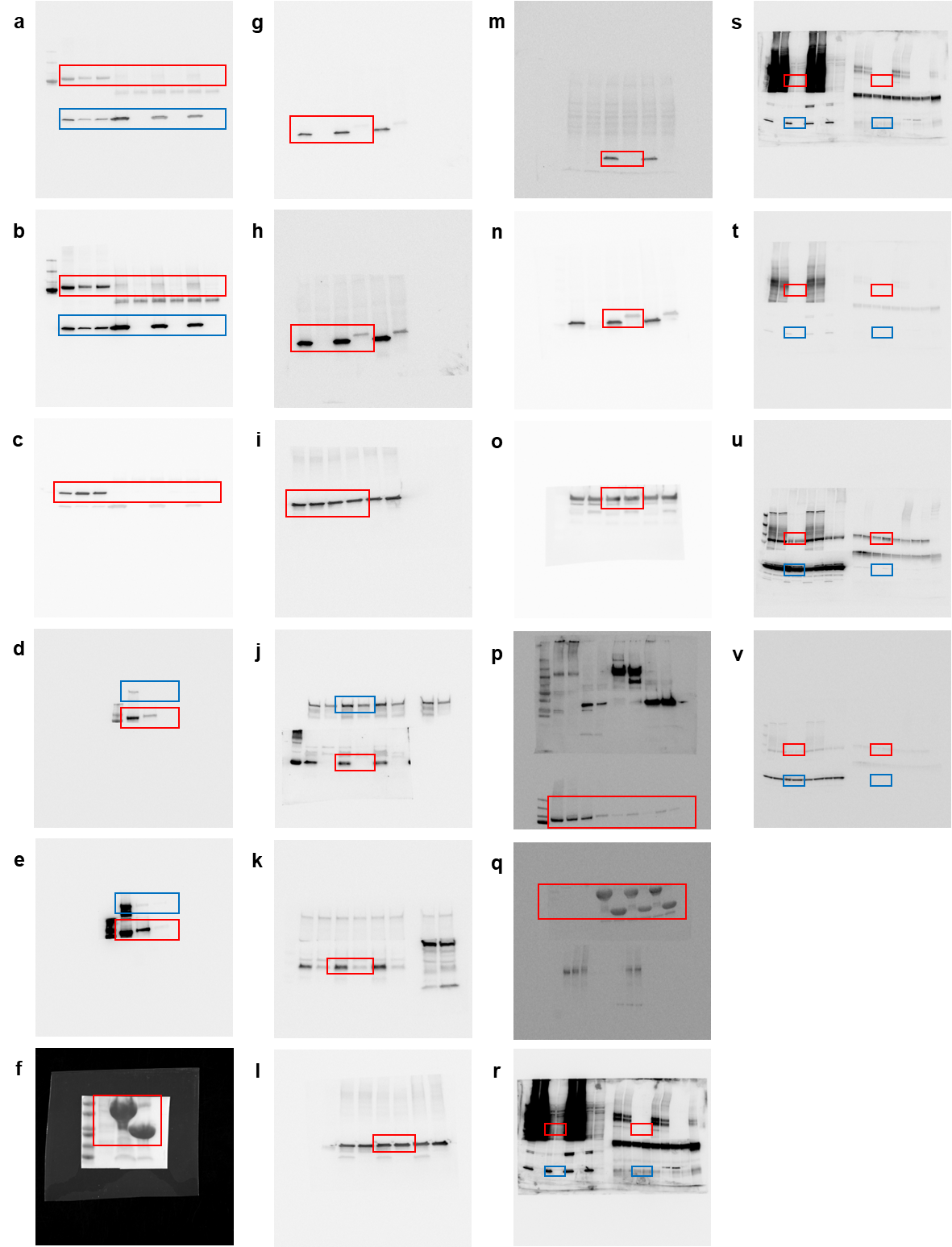


1. Full western blots

Western blots for AGO2 (red) and GRB2 (blue) with, **a**, short and, **b**, long exposure and, **c**, GAPDH, for Fig. 1c. Western blots for AGO2 (red) and DICER1 (blue) with, **d**, short and, **e**, long exposure and, **f**, ponceau stain for GST proteins from Fig. 3a. Western blots for GRB2 with, **g**, short and, **h**, long exposure and, **i**, GAPDH, for Fig. 4a. Western blots for, **j**, DICER1 (blue) and GRB2 (red), **k**, LIN28B, **l**, GAPDH, **m**, HMGA2, **n**, GRB2 and, **o**, α-Tubulin. Western blots for, **p**, AGO2 and, **q**, GST, for Extended Data Fig. 1. Western blots for pY99 (red) and GRB2 (blue) with, **r**, long, **s**, short, and, **t**, very short exposure for Extended Data Fig. 9. Western blots for AGO2 (red) and GAPDH (blue) with, **u**, long and, **v**, short exposure for Extended Data Fig. 9. Bands are indicated by boxes.

**Extended Data Table 1: Sequences and locations of AGO2 peptides used in isothermal titration calorimetry with GRB2**

Prolines in red form canonical proline-rich motifs.

| Peptide | Residues | Location | Sequence |
| --- | --- | --- | --- |
| AGO2 #1 | 1-20 | N-terminal | MYSGAGPALAPPAPPPPIQG |
| AGO2 # 2 | 21-40 | N-termina | YAFKPPPRPDFGTSGRTIKL |
| AGO2 #3 | 317-333 | PAZ domain | KLVLRYPHLPCLQVGQE |

**References**

1. Schirle, N. T., Sheu-Gruttadauria, J. & MacRae, I. J. Structural Basis for microRNA Targeting. *Science* **346**, 608–613 (2014).

2. Altschul, S. F., Gish, W., Miller, W., Myers, E. W. & Lipman, D. J. Basic local alignment search tool. *J. Mol. Biol.* **215**, 403–410 (1990).
